# TWIST1 a New Determinant of Epithelial to Mesenchymal Transition in *EGFR* Mutated Lung Adenocarcinoma

**DOI:** 10.1371/journal.pone.0029954

**Published:** 2012-01-17

**Authors:** Karine Pallier, Anatole Cessot, Jean-Francois Côté, Pierre-Alexandre Just, Aurélie Cazes, Elizabeth Fabre, Claire Danel, Marc Riquet, Mojgan Devouassoux-Shisheboran, Stéphane Ansieau, Alain Puisieux, Pierre Laurent-Puig, Hélène Blons

**Affiliations:** 1 UMR-S775, INSERM, Paris, France; 2 Université Paris Descartes, Paris, France; 3 Department of Pathology, Hôpital Ambroise Paré, AP-HP, Université de Versailles St Quentin en Yvelines, Boulogne-Billancourt, France; 4 Department of Biology, Hôpital Européen Georges Pompidou, AP-HP, Paris, France; 5 Department of Pathology, Hôpital Bichat, AP-HP, Paris, France; 6 Department of Thoracic Surgery, Hôpital Européen Georges Pompidou, AP-HP, Paris, France; 7 Hôpital de la Croix-Rousse, Lyon, France; 8 INSERM, U590, Lyon, France; 9 Centre Léon Bérard, Lyon, France; 10 Université Lyon I, Lyon, France; University of Colorado, Boulder, United States of America

## Abstract

Metastasis is a multistep process and the main cause of mortality in lung cancer patients. We previously showed that *EGFR* mutations were associated with a copy number gain at a locus encompassing the *TWIST1* gene on chromosome 7. *TWIST1* is a highly conserved developmental gene involved in embryogenesis that may be reactivated in cancers promoting both malignant conversion and cancer progression through an epithelial to mesenchymal transition (EMT). The aim of this study was to investigate the possible implication of TWIST1 reactivation on the acquisition of a mesenchymal phenotype in *EGFR* mutated lung cancer. We studied a series of consecutive lung adenocarcinoma from Caucasian non-smokers for which surgical frozen samples were available (n = 33) and showed that TWIST1 expression was linked to *EGFR* mutations (P<0.001), to low CDH1 expression (P<0.05) and low disease free survival (P = 0.044). To validate that TWIST1 is a driver of EMT in *EGFR* mutated lung cancer, we used five human lung cancer cell lines and demonstrated that EMT and the associated cell mobility were dependent upon TWIST1 expression in cells with *EGFR* mutation. Moreover a decrease of EGFR pathway stimulation through EGF retrieval or an inhibition of TWIST1 expression by small RNA technology reversed the phenomenon. Collectively, our *in vivo* and *in vitro* findings support that TWIST1 collaborates with the EGF pathway in promoting EMT in EGFR mutated lung adenocarcinoma and that large series of *EGFR* mutated lung cancer patients are needed to further define the prognostic role of TWIST1 reactivation in this subgroup.

## Introduction

Lung cancer is the most common cause of cancer death in western countries. Tumor recurrence and metastasis are frequent events despite the establishment of multiple lines of therapy and the introduction of targeted agents [1]. Tyrosine kinase inhibitors against the epidermal growth factor receptor (EGFR) have been developed and although the vast majority of patients with lung cancer failed to respond, a minority showed dramatic tumor shrinkage. Molecular screenings showed that the presence of an EGFR activating mutation was highly linked to tumor response and *EGFR* mutated tumors were defined as a new entity among lung cancers [2]. Two alterations account for more than 90% of the mutants; inframe deletions in exon 19 and the p.L858R missense mutation that were both shown to activate the EGFR pathway mainly through PI3K/AKT and STAT3 activation and to remain at least partially ligand dependent. *EGFR* mutations are more common in East Asians, in non-smokers, in women and in patients with adenocarcinomas (ADC) especially those with bronchioalveolar (BAC) features. Although it is largely accepted that patients with *EGFR* mutated tumors have a better prognosis as compared others, aggressive tumors with rapid unfavorable evolution also exist. Prognostic markers have not been specifically studied in *EGFR* mutated cancers and could help classify this new entity [3]. In a previous work, using SNP array, we showed that tumors with *EGFR* mutations had a copy number increase of a region on chromosome 7 (7p21.1-7p15.3) encompassing the *TWIST1* gene, a highly conserved basic helix-loop-helix transcription factor regulator of embryogenesis [4]. This was confirmed on CGH arrays with 40% of *EGFR* mutated tumors showing copy number increase of this region. In order to go further we focused on TWIST1 and explored whether it could be the potential target. While TWIST1 is silent in most healthy adult tissues, it was found overexpressed in various types of carcinomas [5], [6], [7], [8], [9], [10], [11], [12], [13]. Described as a pro-metastatic factor, TWIST1 was found to promote cell motility and invasiveness through an epithelial-mesenchymal transition (EMT) [14]. In epithelial cells, EMT induction was related to cooperation between TWIST1 and mitogenic proteins such as ERBB2 and RAS. TWIST1 was additionally found to override oncogene-induced senescence and apoptosis by interfering with both p53 and RB. Therefore both roles on senescence and oncogenic pathways could drive cell transformation [15], [16]. In this work, we tested the hypothesis that TWIST1 and mutated EGFR could similarly cooperate in promoting EMT in lung adenocarcinomas. Our results suggest that TWIST1 is an important driver of EMT in *EGFR* mutated cells and could be a potential marker in clinics to predict outcome in patients with *EGFR* mutated tumors.

## Results

### TWIST1 expression is associated with *EGFR* mutation in lung cancer

**Figure 1:**
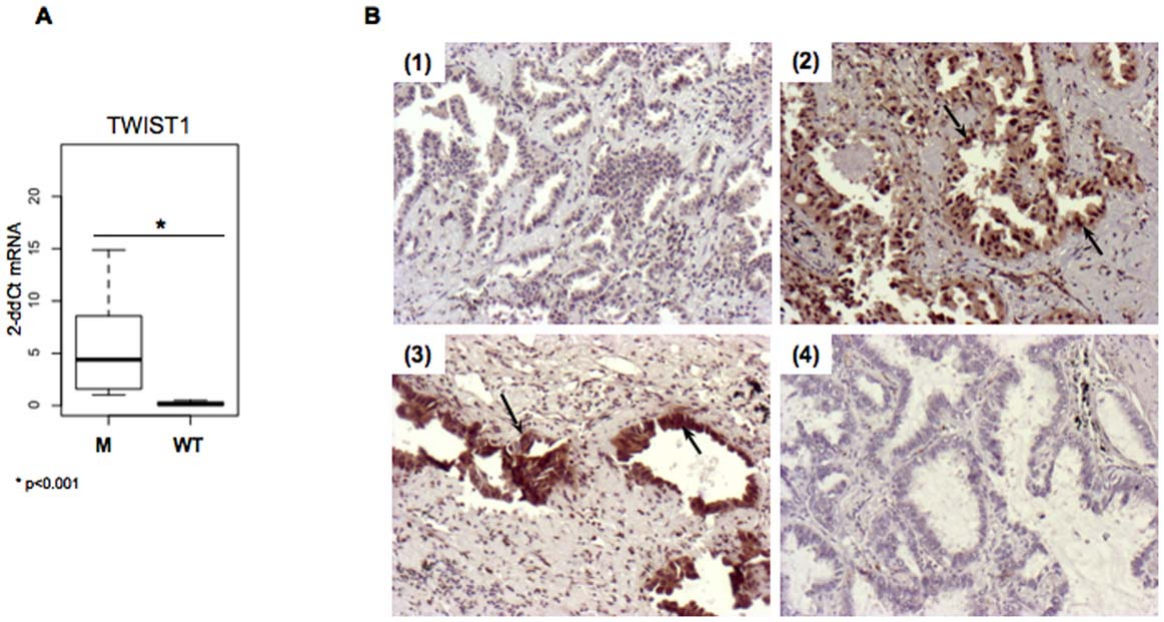
TWIST1 expression in tumor samples. **A.** Assessment of *TWIST1* expression by quantitative RT-PCR in *EGFR* mutated (M) and wild type (WT) tumors assessed by quantitative RT-PCR. **B.** Representative microphotographs (10×) of immunohistochemical expression of TWIST1 in primary *EGFR* mutated human lung tumors (sample 230 (1), sample 55 (2), sample 288 (3)) or wild type tumors (sample 149 (4)). Arrows point out cells for which staining is positive. TWIST1 positive staining is seen for samples 2 and 3 in cytoplasm and nucleus.

The first objective of this work was to examine whether TWIST1 reactivation could be associated to *EGFR* mutation. Therefore we compared TWIST1 expression, at mRNA and protein levels, to the *EGFR* mutational status in a group of tumors from non-smokers. Among a series of 213 Caucasian patients with lung ADC, 33 had not smoked or less than 10 pack-year and were selected for this study. Twenty-two tumors had high quality RNA available with RQI>7, 30 had Formalin Fixed Paraffin Embedded (FFPE) tissue for Immuno-Histo-Chemistry analyses (IHC) and 21 were *EGFR* mutated (Table S1). In this group of non-smokers high levels of *TWIST1* mRNA were found in *EGFR* mutated tumors (p<0.001) (Fig. 1A). Moreover *TWIST1* mRNA correlated with TWIST1 positive staining by IHC (p = 0.0012). IHC showed that staining was nuclear and cytoplasmic and restricted to tumor glands (Fig. 1B). We further explored a potential interplay between TWIST1 reactivation and EMT in non-smoker lung cancer. Considering mRNA and protein information, TWIST1 was positive in only one *EGFR* wild type tumor (sample 133). This sample is an adenosquamous carcinoma it is the only particular non-ADC or BAC in this series.

**Figure 2:**
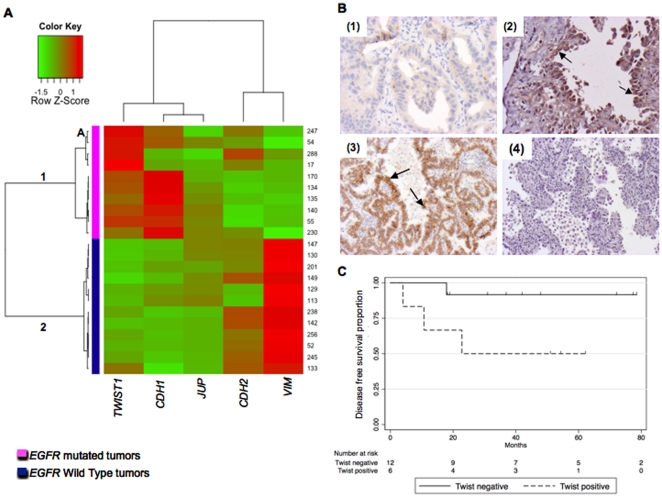
TWIST1 and CDH1 expression are inversely correlated in human lung primary tumors. **A.** *TWIST1*, *CDH1*, *JUP*, *CDH2* and *VIM* expressions were assessed by quantitative RT-PCR in human lung primary tumors. Unsupervised hierarchical clustering isolated two groups. *EGFR* mutated tumors were classified in group 1 and *EGFR* wild type tumors in group 2. Group 1A, contains EGFR mutated TWIST1 reactivated tumors (247, 54, 17 and 288) with an intermediate phenotype (high *TWIST1*, low *CDH1*, low VIM expression) that could be undergoing an epithelial to mesenchymal transition. **B.** Representative microphotographs (20×) of immunohistochemical expression of CDH1 (1, 3) and TWIST1 (2, 4) in *EGFR* mutated tumors. Positive cells are indicated by arrows. Upper panels: example of tumor (sample 17), expressing a low amount of CDH1 (1) and TWIST1 (2). Lower panels: example of tumor (sample 18) expressing CDH1 (3) and lacking TWIST1 (4). **C.** Kaplan-Meier Disease free survival curves according to TWIST1 reactivation assessed by IHC on lung tumor tissue samples. Disease free survival is shortened in the TWIST1 positive group (p = 0.044).

Unsupervised hierarchical clustering based on *CDH1*, *TWIST1*, *CDH2*, *VIM* and *JUP* mRNA levels showed perfect distinction of groups according to the *EGFR* mutational status (Fig. 2A). Patterns of expression were mostly mesenchymal for the wild type *EGFR* tumors suggesting that EMT is not dependent upon TWIST1 in this group. In the *EGFR* mutated group, although patterns of expression were in majority epithelial, low CDH1 correlated with TWIST1 reactivation at mRNA (p<0.05) (group 1A) and protein levels (0.017) (Fig. 2B). In order to go further, *SNAI1 (SNAIL1)* and *ZEB1* were analyzed at RNA level. No *ZEB1* reactivation was seen in either group of tumors but 2 samples, one *EGFR* mutated (sample 288) and one *EGFR* wild type (sample 245) showed *SNAI1* reactivation. The *EGFR* mutated *SNAI1* reactivated tumor express *TWIST1*, *VIM* and *CDH2* (sample 288) suggesting that TWIST1 and *SNAI1* might interact to drive full mesenchymal phenotype (Table S1).

Furthermore, in patients with *EGFR* mutated tumors disease free survival was shorter for patient with TWIST1 reactivated tumors (IHC positive samples) and reach significance although the number of patients (n = 21) is low and deserve confirmation (p = 0.044).

Collectively, these observations support the assumption that TWIST1 could promote EMT in *EGFR* mutated tumors. In order to validate these observations we examined whether TWIST1 and EGFR proteins cooperate in promoting EMT and the associated mobility in human lung cell lines.

### Assessment of TWIST1 expression in lung adenocarcinoma cell lines

**Figure 3:**
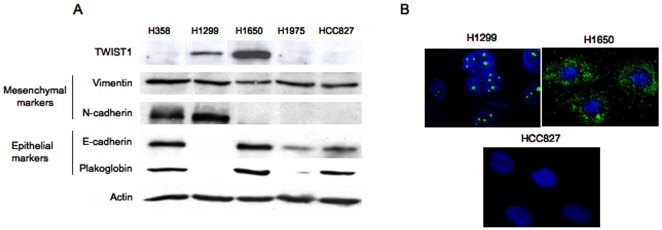
Basal expression of EMT markers in cell lines. **A.** Assessment of TWIST1, Vimentin, N-Cadherin, E-Cadherin and Plakoglobin showing qualitative expression of markers by western blotting. **B.** Representative photomicrographs of TWIST1 expression in *EGFR* mutated cell lines (H1650 and HCC827) and in H1299 as assessed by immunofluorescence.

**Figure 4:**
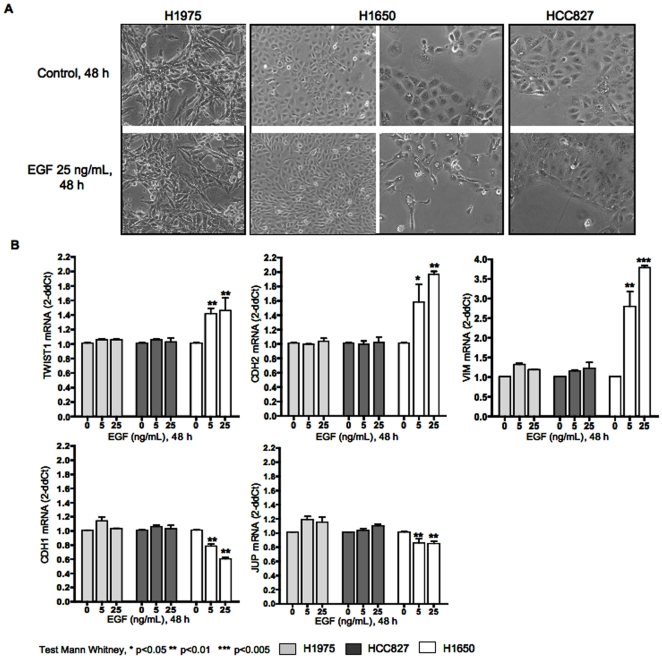
EGF induces EMT only in cells with *EGFR* mutation and *TWIST1* expression. EGFR mutated human lung cancer cell lines (H1975, H1650, HCC827) were treated with EGF. **A.** Cell morphology obtained by phase-contrast microscopy. **B.** *TWIST1*, *CDH2*, *VIM*, *CDH1* and *JUP* expression as assessed by quantitative RT-PCR. mRNA levels are expressed relative to the untreated control condition. Each column represents the mean ±SD of 3 independent experiments each one done in triplicate.

**Figure 5:**
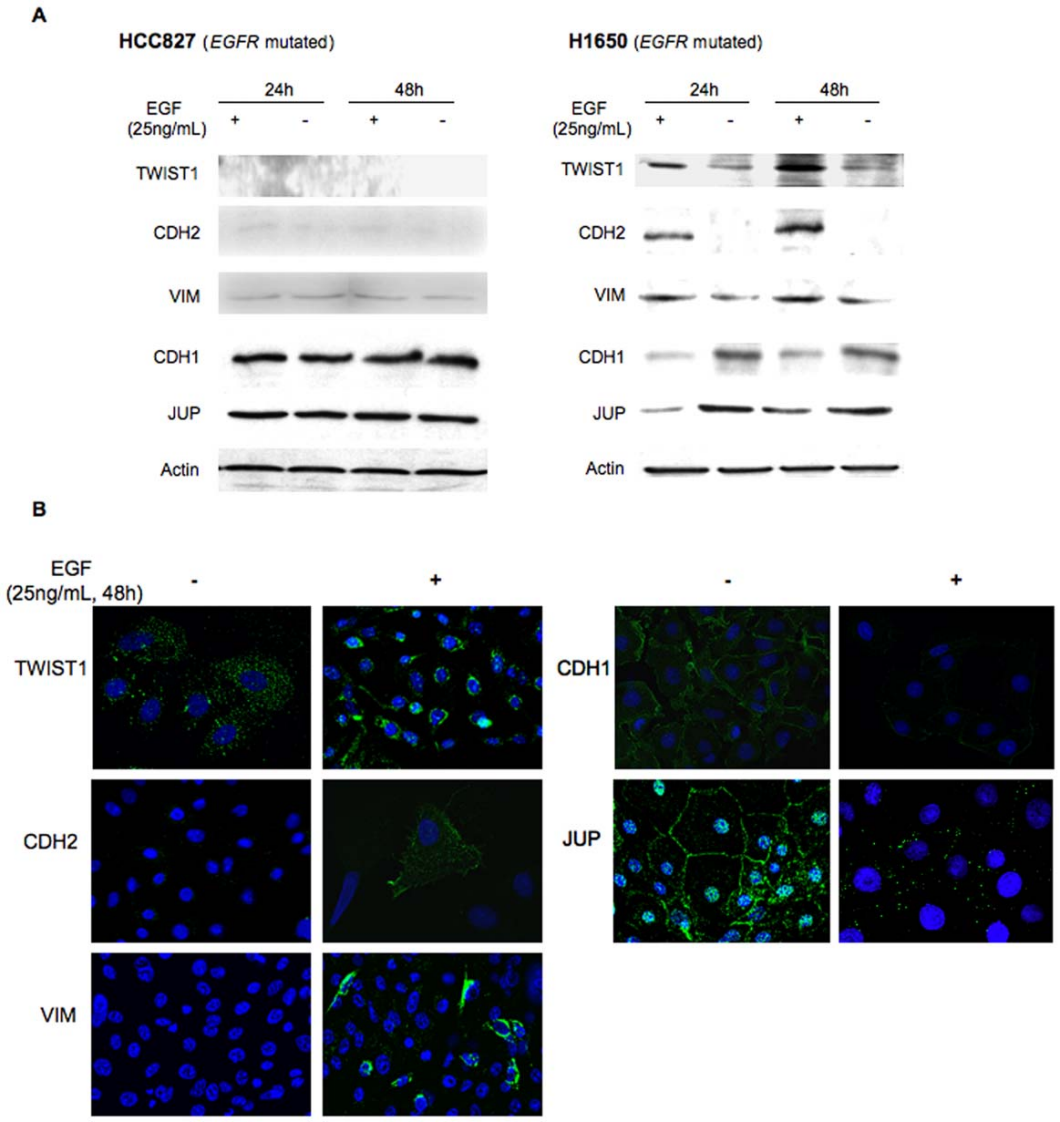
EGF induces EMT only in cells with *EGFR* mutation and *TWIST1* expression (protein level). **A.** Assessment of TWIST1, CDH2, VIM, CDH1 and JUP expression by western blotting after addition of EGF for a 24 h or 48 h period of time on HCC827 (*EGFR* mutated, TWIST1 not expressed) and H1650 (*EGFR* mutated, TWIST1 expressed). **B.** Representative photomicrographs of TWIST1, CDH2, VIM, CDH1 and JUP expression analyzed by immunofluorescence after EGF treatment (25 ng/mL).

We examined whether TWIST1 reactivation was associated with mutated *EGFR* in human lung carcinoma cell lines and related to a mesenchymal phenotype. We first assessed *TWIST1* mRNA expression in 5 cell lines including two EGFR wild type: H358 (KRAS mutated, unknown smoking status) and H1299 (*NRAS* mutated, smoker) and three *EGFR* mutated isolated from non-smokers: H1650, H1975 and HCC827. H1650, H1975 and HCC827 harbor classic *EGFR* mutations that can be compared to the mutations found in our series of patients (an in-frame deletion in exon 19 for H1650 and HCC827 and the L858R mutation for H1975). H1299 and H1650 were the only ones with high *TWIST1* levels as compared to normal human lung RNA used as calibrator (Table 1). Consistently, TWIST1 protein was present in these two cell-lines. These results show that, as in tumors, TWIST1 reactivation was found in lung cancer cell lines, one is *EGFR* mutated and one is *NRAS* mutated they were established from a non-smoker and a smoker respectively (Table 1). Light microscopy examination showed that H1299 cells displayed a mesenchymal phenotype whereas H1650 cells presented an epithelial morphology (Fig. S1). TWIST1 was localized in the cytoplasm in H1650 cells and strictly located in nucleoli in H1299 cells (Fig. 3 A–B). This observation suggested that signals essential for TWIST1 nuclear localization could be lacking in H1650. Indeed H1299 cells are *NRAS* mutated (p.Q61K) and have a constitutive activation of both MAPK and PI3K downstream pathways. As *EGFR* activating mutations are known to remain at least partially ligand dependent in inducing oncogenic signals [2], we next examined whether EGFR activation by EGF might induce TWIST1, restore its nuclear localization and contribute to the induction of EMT. H1650 cells were treated during 4, 12, 24 and 48 hours with EGF at concentrations ranging from 5 to 120 ng/ml. First modifications in mRNA expression occurred at 24 h, 5 ng/ml (data not showed). All cells were therefore treated at 5 and 25 ng/ml during 24 and 48 hours in triplicate and three independent experiments. In TWIST1 reactivated *EGFR* mutant cells (H1650), EGF was sufficient to induce TWIST1 nuclear translocation and to promote EMT as indicated by cell morphologic changes and by the expected shift from epithelial to mesenchymal markers (Fig. 4 and 5). In cell lines without TWIST1 reactivation, H358, HCC827 or H1975, EGF did not modify cell phenotype although two are *EGFR* mutated. In these cell lines, at basal state phenotypes can be complex with *VIM* and *CDH2* expression for H1975 and H358 and a true epithelial phenotype for HCC827. The analysis of two other EMT transcription factors, *ZEB1* and *SNAI1* demonstrated no reactivation of *ZEB1* but overexpression of *SNAI1* in H1975 that could explain the mesenchymal could explain the mesenchymal aspect of these cells at basal state (Table 1; Fig. S1). No up-regulation of SNAI1 was seen after EFG treatment in this cell line.

**Table 1:** Expression of *TWIST1*, mesenchymal (*CDH2*, *VIM*, *SNAI1*, *ZEB1*) and epithelial (*CDH1*, *JUP*) markers in lung adenocarcinoma cell lines.

	*EGFR* mutated	*TWIST1*	Mesenchymal markers				Epithelial markers	
			*CDH2*	*VIM*	*SNAI1*	*ZEB1*	*CDH1*	*JUP*
**H1299**	**No**	13.21±0.98	10.90±0.09	1.73±0.05	0.12±0.02	0.43±0.13	0.01±0.02	0.01±0.04
**H358**	**No**	0.08±0.04	13.01±0.12	0.15±0.02	2.04±0.19	0.02±0.01	6.17±0.79	1.80±0.34
**H1650**	**p.E746-A750del**	12.37±0.94	0.76±0.03	0.01±0.36	1.32±0.12	0.01±0.02	11.74±0.20	2.95±0.44
**H1975**	**p.L858R/p.T790M**	0.04±0.12	0.12±0.07	1.45±0.04	5.39±0.22	0.31±0.09	2.85±0.26	0.95±0.05
**HCC827**	**p.E746-A750del**	0.19±0.10	0.70±0.10	0.10±0.34	0.23±0.14	0.04±0.07	6.51±1.01	2.30±0.32

Assessment of *TWIST1*, *CDH2* (N-Cadherin), *VIM* (Vimentin), *SNAI1 (SNAIL1)*, *ZEB1*, *CDH1* (E-Cadherin) and *JUP (plakoglobin)* transcriptional expression by quantitative RT-PCR. The relative expression levels between samples were calculated using the comparative delta Ct (threshold cycle number). In each run, in addition to study samples, normal human lung RNA was used as calibrator. Each value represents the mean ±SD of three independent experiments each one run in triplicate.

To further study the influence of TWIST1 reactivation in a background of EGFR mutation we focused our work on HCC827 (TWIST-) and H1650 (TWIST+), both are epithelial at basal state, harbor similar EGFR mutation and were derived from non-smokers. To explore whether TWIST1 is a prerequisite for EGF induced EMT in *EGFR* mutated lung cancer cells, we confirmed that long time EGF treatment (10 days) did not generate an EMT in HCC827 TWIST1 negative cells. At the opposite HCC827 switched to a mesenchymal phenotype in response to TGFβ. This TGFβ induced EMT was independent of TWIST1 reactivation strengthening the role of TWIST1 in specifically driving an EGF/EGFR-induced EMT (Fig. S2).

EMT is known to be a reversible process subjected to microenvironmental changes. We therefore sought to explore whether TWIST1-dependent EMT might similarly be reversed when EGF is released in H1650 cells. Following EGF withdraw, cells reverted to an epithelial phenotype as indicated by morphological changes and the expected shift from mesenchymal to epithelial markers (Fig. S3). Collectively, these results strongly support the assumption that EGFR activation contributes to TWIST1 re-localization and thereby promotes EMT.

### TWIST1 depletion alleviates EGF induced EMT in *EGFR* mutated-TWIST1 expressing cells (H1650)

**Figure 6:**
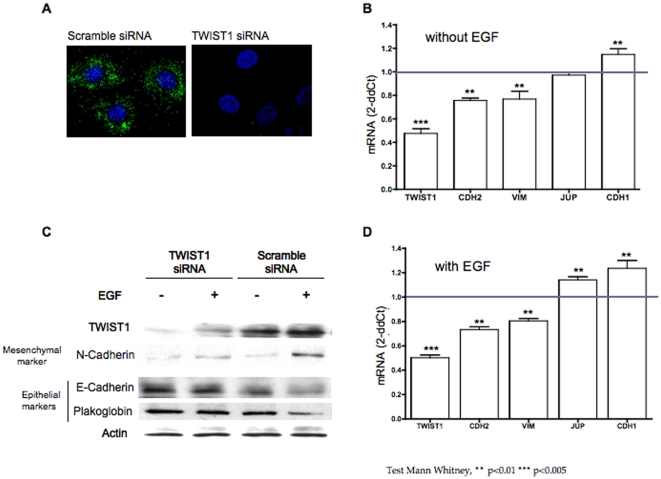
TWIST1 depletion through RNA interference overrides EMT induction by EGF in H1650 cells. *EGFR* mutated, TWIST1 expressed H1650 human lung cells were depleted of TWIST1 through RNA interference. Impact of TWIST1 depletion on epithelial and mesenchymal markers in absence or in presence of EGF was examined. **A.** The efficiency of the siRNA was controlled by assessing TWIST1 expression by immunofluorescence. **B.** Expression analysis of *TWIST1*, *CDH2*, *VIM*, *CDH1* and *JUP* in absence of EGF treatment by quantitative RT-PCR. Graph shows the relative expression (siRNA TWIST1/siRNA scramble) for the 5 markers. Each column represents the mean ±SD of 3 independent experiments each one done in triplicate. **C.** Impact of TWIST1 depletion on TWIST1, CDH2, CDH1 and JUP protein expression in H1650 cells in absence or in presence of EGF as assessed by western blotting. **D.** Impact of TWIST1 depletion on *TWIST1*, *CDH2*, *VIM*, *CDH1* and *VIM* following cell treatment with EGF as assessed by relative expression (siRNA TWIST1/siRNA scramble). Each column represents the mean ±SD of 3 independent experiments each one done in triplicate.

TWIST1 depletion through RNA interference in H1650 cells resulted in a slight reduction of mesenchymal markers (*CDH2* and *VIM*) and induction of CDH1 in absence of EGF. This result was somehow expected as we previously showed that TWIST1 was localized in the cytoplasm and might not be fully active at basal state. TWIST1 depletion also resulted in *SNAI1* and *ZEB1* mRNA decrease (data not showed). Furthermore, TWIST1 depletion counteract EGF induced EMT in *EGFR* mutated-TWIST1 expressing cells (Fig. 6 A–D). Finally TWIST1 depleted cells maintained an epithelial phenotype under EGF treatment.

### EGFR-TWIST1-induced EMT is associated with a gain in cellular motility

**Figure 7:**
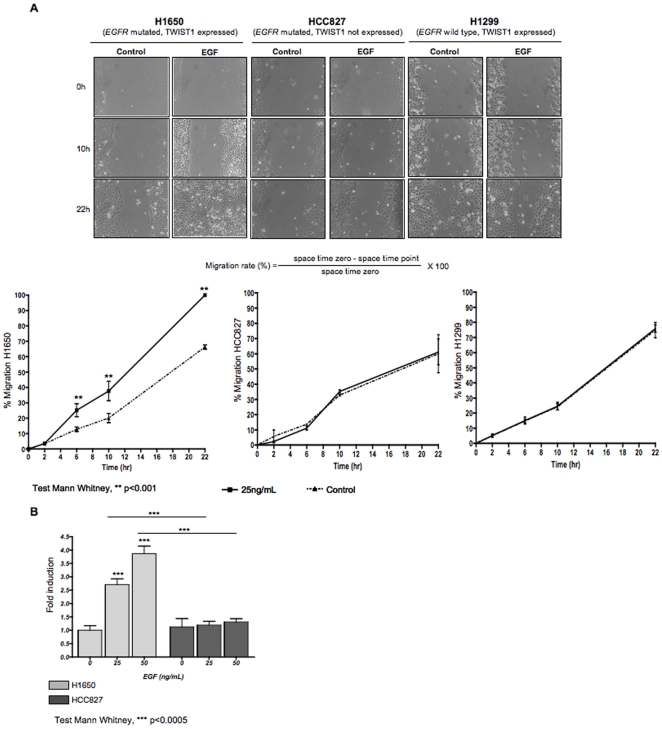
EGF-induced EMT associates with a gain in motility. Human lung cell lines harboring different TWIST1 and EGFR status were treated with EGF and tested for their motility in scratch (panel A) and transwell migration (panel B) assays. A. Phase contrast photographs showing wound closure for H1650, HCC827 and H1299 cell lines in presence or absence of EGF and corresponding graphs presenting the mean ±SD of wound closure in 3 independent experiments each one run in triplicate. B. Migration potential assessed by transwell migration. Graphs show the ratio: number of cells that migrated in the lower chamber in the EGF conditions reported to number of cells that migrated in the lower chamber in absence of EGF treatment. EGF only enhanced migration in cells with EGFR mutation and TWIST1 reactivation.

As EMT is known to drive cell dissemination, we next explored whether EGF treatment provides cells with motility. To this end, H1650 (*EGFR* mutated, TWIST1 reactivated), H1299 (*EGFR* wild type, TWIST1 reactivated) and HCC827 (*EGFR* mutated, TWIST1 absent) cell lines were treated with EGF and their motility was assessed in a scratch assay. As shown in Fig. 7, EGF treatment resulted in a significant motility gain in H1650 cells (38% versus 20% after 10 h (p<0.001) and 100% versus 66% wound closure after 22 h (p<0.001)). These results strengthened our conclusion that EGFR activation through its ligand is essential in promoting TWIST1-associated EMT. As expected, EGF did not impact on HCC827 and H1299 motility (Fig. 7A). Similar results were obtained when motility was assessed in a migration assay (Fig. 7B).

These results showed that EGF strictly controlled the EMT-associated motile phenotype in EGFR mutated and TWIST1 reactivated cells.

Collectively our results suggest the existence of a cooperative effect between EGF pathway activation and TWIST1 reactivation in promoting EMT in *EGFR* mutated lung cancer.

## Discussion

*EGFR* mutations are specific to lung cancer and occur in mainly non-smokers. This specific subgroup was extensively studied because of the dramatic sensibility of *EGFR* mutated cells to anti-EGFR tyrosine kinase inhibitors (TKI) but the understanding of tobacco independent lung carcinogenesis is far from being totally understood. We found that *TWIST1*, an embryonic transcription factor actor of the metastatic process [17], was specifically expressed in tumors with *EGFR* mutations in a group of tumors from non-smoker. In one series of Taiwanese patients with lung cancer TWIST1 expression was shown in 36% of samples but the relation with *EGFR* mutational status was not reported [18]. Because TWIST1 and mitogenic proteins (ie ERBB2, RAS) were found to cooperate in EMT induction [15], we investigated whether TWIST1 could trigger a mesenchymal phenotype in *EGFR* mutated cells.

Consistent with the literature, in our series, most lung tumors with *EGFR* mutations were associated with epithelial characteristics [19]. Tumors that were not *EGFR* mutated demonstrated higher levels of mesenchymal markers independently of TWIST1 reactivation. Recent findings from Takeyama *et al.* in lung cancer cells lines demonstrated that among four master epithelial-to-mesenchymal transition (EMT)-inducing genes (*ZEB1*, *SIP1*, *SNAI1*, and *SNAI2* (*SLUG*)) *ZEB1* was highly expressed in non-EGFR mutated mesenchymal cell lines whereas *EGFR* mutated cell lines had low ZEB1 expression and an epithelial phenotype [20]. In our experience non-EGFR mutated human tumors from non-smokers express mesenchymal markers but *ZEB1* could not be linked to this phenotype suggesting that other transcription factors might be implicated. Within the *EGFR* mutated one, high TWIST1 expression was related to a significant reduced CDH1 expression, suggesting that among those epithelial tumors, a subset could have started an EMT program. For these samples, tissue analysis by the pathologist did not identify any change such as fibroblastic transformation but morphologic examination of tissues rarely recognizes mesenchymal features on tissue slides. Indeed, EMT is a dynamic continuous event that may be difficult to characterize in tumors because cells might not be at the same state at the same time leading to tumor heterogeneity [21]. In order to investigate whether TWIST1 could determine morphological changes in *EGFR* mutated tumors we analyzed the effect of TWIST1 expression *in vitro* and showed that in *EGFR* mutated lung cancer cells, EGF pathway stimulation and TWIST1 cooperated to induce an EMT and the associated cell mobility. In other tumor models, EGF was shown to trigger an EMT with up-regulation of either SNAI1 or TWIST1 possibly through STAT3 activation [8], [22]. In our study EGF does not dramatically enhance TWIST1 expression but is more likely to induce its nuclear re-localization that associated with the gain of a mesenchymal phenotype. In this model the loss of CDH1 expression is only an indirect proof but suggests that TWIST1 might directly bind DNA to induce EMT. Post-translational regulation of TWIST1 might also be mediated through mutated EGFR activation, indeed, in head and neck cancer, IL6 activation was recently shown to stabilize TWIST1 through CK2 phosphorylation [23]. The mutated *EGFR*/TWIST1 interdependence was validated using RNA silencing. TWIST1 depletion in other cancers such as prostate and gastric carcinoma also counteract the EMT program [6]. In our model TWIST1 expression also favored cell mobility and could therefore enhance the metastatic potential of tumor cells. Other transcription factors have been implicated in the acquisition of an EMT such as ZEB1, E47, SNAI1, and *SNAI2* [24]. In our experience *ZEB1* and *SNAI1* were slightly increased in H1650 cells after EGF treatment and *SNAI2* was up-regulated at protein level suggesting a possible cooperation between transcription factors. This was not retrieved for the TWIST1 non-expressing *EGFR* mutated cells in which *ZEB1* and *SNAI1* remained at basal state (figure S4). Here we show that in EGF treated *EGFR*-mutated cells, *ZEB1* and *SNAI1* up-regulation seems related to TWIST1 reactivation but other mechanism could drive EMT in absence of EGF stimulation. Indeed *SNAI1* reactivation was found independently of *TWIST1* in H1975 and the mesenchymal aspect of these cells is independent of EGF stimulation.

Finally, cancer cells undergoing EMT need to reverse through mesenchymal-epithelial transition (MET) when settled in their new environment [21]. In our model, we confirmed that EGF-driven EMT was reversible in *EGFR* mutated TWIST1 expressing lung cancer cells.

In alternative models TWIST proteins were also shown to override premature senescence due to mitogenic activities of oncoproteins [15]. In our experience, TWIST1 depletion using high siRNA concentration (>100 pmol) led to cell death suggesting that TWIST1 retrieval allowed cell death to occur in those cells. TWIST1 effects on cell death could rely on its relation with the p53 pathway via an inhibition of *ARF* mRNA expression. We had previously found an association between *ARF-CDKN2A* homozygous deletions and *EGFR* mutations [4]. In this study, 5 tumors had an *ARF-CDKN2A* homozygous deletion and none reactivated TWIST1. Also work is needed to validate this finding we can suspect that in tumors without *ARF-CDKN2A* deletion TWIST1 reactivation could override oncogene premature senescence by interacting with CDKN2A (Table S1). Finally EMT has previously been implicated in resistance to chemotherapy [25] and because TWIST1 may be linked to cell death inhibition its role on sensitivity to TKI should be further investigated.

Collectively our results report for the first time that EGF induced EMT in *EGFR* mutated lung tumors is driven by TWIST1 reactivation. Indeed the transition from epithelial to mesenchymal phenotype as well as the associated increased mobility is dependent upon both TWIST1 and EGF activation *in vitro*. This *in vitro* model is coherent with our *in vivo* observations. Moreover TWIST1 in tumors could also counteract oncogene-induced senescence through *ARF-CDKN2A*. Although further work is warranted to confirm this finding our results show that even in localized lung tumors TWIST1 may be reactivated and is associated with EMT. This work supports the idea that in *EGFR* mutated lung tumors TWIST1 reactivation could promotes progression through EMT induction and needs to be evaluated in prospective series as a new prognosis marker.

## Materials and Methods

### Ethic Statements

Tumors were retrospectively selected in the European Georges Pompidou hospital tumor bank in accordance to French laws from patients that had specified orally or by written consent to the surgeon that they were not opposed to the use of their surgical tumor samples for research purpose. Patients had finished their treatment at collection time. Tissue bank and annotations were anonymized and no connection between clinics and research was possible.

Our study was subjected to the ethic committee “Comité de Protection des Personnes, Ile de France II” (Committee for the Protection of People) linked to the Paris Descartes University that qualified the 213 clinical specimens for research use (N°CPP Ile de France II 2008-136). Moreover, the collection was declared at and approved by the Research and Education Ministry, DC-2008-401, N°ID-RCB: 2008-A00880-55.

### Case selection

Primary lung cancer were prospectively collected and stored frozen at time of surgery from 2004 to 2006 in the Georges Pompidou European hospital in Paris. Among the consecutive adenocarcinoma cases (n = 213), 33 were non-smokers. These tumors had previously been characterized for mutation testing at *EGFR*, *TP53*, *KRAS*, *BRAF*, *PI3KCA*, *STK11*, *ERBB2* and *AKT1* and for *CDKN2A* homozygous deletion table S1 [4].

Briefly, patients had surgery as first line treatment for NSCLC and adjuvant treatment if necessary following standardized guidelines. Survival data were retrospectively collected and time to first relapse post surgery was considered for relapse free survival analysis.

### RNA extraction and Real-Time Reverse Transcriptase-PCR

Total RNAs from cell lines and frozen tumor samples, 100 slices (7 µm) cut on a cryostat, were extracted using RNeasy® Mini kit (Qiagen). Two HES slides were done at the beginning and the end of tissue slices confirming the existence of more than 70% of tumors cells. RNA quality was estimated by the RQI (RNA quality indicator) using high-resolution electrophoresis system (Experion™, Biorad, Paris).

RNAs (2 µg) were reversed-transcribed using the Reverse Transcription Kit (Applied Biosystem). mRNA levels of *TWIST1*, *CDH1*, *CDH2*, *JUP*, *SNAI1*, *ZEB1* and *VIM* were quantified by SYBR green real-time polymerase chain reaction (PCR) on an ABI Prism 7900 sequence detector system (Applied Biosystems, Foster City, CA). Real-time PCR was performed in triplicate reactions with 20 ng of cDNA using SYBR Green PCR Master Mix (Applied Biosystems). In each run, normal human lung RNA (Clontech) was used as calibrator. We used *POLR2A* (Polymerase RNA II polypeptide A), *RPL13A* (Ribosomal Protein L13A), *YAP1* (Yes-Associated Protein 1) and *18S* RNAs as the endogenous genes controls. The relative expression levels between samples were calculated as described by Livak et al. [26]. All experiments were run in triplicates and in 3 independent PCR experiments. Primer sequences are detailed in Table S2.

### Cell culture experiments

Lung adenocarcinoma cell lines (H358, H1299, H1650, H1975 and HCC827) were obtained from the American Type Culture Collection (ATCC) and grown at 37°C, 5% CO2 in RPMI 1640 supplemented with 10% fetal bovine serum (PAA). Before EGF treatments, cells were serum starved for 24 h then EGF (Invitrogen) was supplemented to 2% foetal bovin serum culture media at two concentrations (5 ng/mL and 25 ng/mL) and treatment times (24 h and 48 h).

### TWIST1 small interfering RNA experiment

Cells were seeded in six-well culture plates in culture medium supplemented with 10% foetal bovine serum. The next day, transfection was done with either TWIST1 or scrambled siRNAs at concentration ranging from 20 to 100 ρmoles (siRNA TWIST1, Santa Cruz). Co-treatment with EGF was done as follow: after 6 h of contact with siRNA transfection reagents, EGF was added to the transfection media (5 or 25 ng/mL) for 24 h and treatment was expanded 24 h more with EGF containing medium alone. Cellular morphology was observed and mRNAs were collected.

### Protein isolation and western blots

Proteins were extracted in RIPA buffer. Total protein extracts (30 or 40 µg/lane) were subjected to analysis protein expression was examined using TWIST1 (Abcam), VIM (Santa Cruz) and JUP (Santa Cruz) monoclonal antibodies and Actin (Sigma), CDH1 (Cell Signaling) and CDH2 (Santa Cruz) polyclonal antibodies. Blots were revealed with horseradish peroxydase-conjugated secondary antibodies (Dako) and ECL Detection Kit (Amersham).

### Immunofluorescence and deconvolution microscopy

Cells were seeded onto glass slides 24 h prior to treatments with EGF. Cells were then treated with EGF, fixed in cold methanol, washed with PBS and permeabilized with saponin. They were incubated with similar primary antibodies as for western blots. Secondary antibodies marked with FITC (Jackson ImmunoResearch) were added for 1 h at room temperature. Cells were mounted in medium with DAPI to label nuclei. After mounting, cells were examined with 40× oil immersion objective (Nikon Eclipse TE-2000E) and AutoQuant X software (MediaCyberrntic) was used to deconvolve z series images.

### Migration assays

Two-dimensional migration assay was performed using a scratch wound model. Migration status was analyzed by residual wound measure at 0 h, 2 h, 6 h, 10 h and 22 h using an inverted phase contrast microscope equipped with a digital camera (Nikon Digital shot DS-L1).

Three-dimensional cell migration assay was performed using the ThinCert™ cell culture insert 12-well plates composed of polycarbonate membrane containing 8 µm pores (Greiner bio-one). Cells mobility is estimated by the number of cells contained in the lower chamber quantified by numeration in Malassez counting chamber.

### Immunohistochemistry

After HES review, 33 tumors were available for immunohistochemistry (IHC). 4 µm-thick sections were deparaffinized using xylene for CDH1 (Cell Signaling) and toluene for TWIST1 (Abcam). CDH1 was performed with an automatic immunostainer Bond III (Leica, Wetzlar, Germany). TWIST1 staining was performed manually. Antigen retrieval was achieved by microwave in citrate buffer pH 6.0. Then, we used the kit UltraVision LP detection (Thermo Scientific). The immunostained sections were examined microscopically by two independent pathologists not aware of the patients' clinical data.

### Statistical analyses

All the data are representative of at least three experiments. Values are expressed as mean±sd. Qualitative variables were tested using Mann-Whitney test and Fisher's exact test. Statistical analysis was carried out using GraphPad Prism 4.0 software (GraphPad Prism Software, Inc., San Diego, CA), STATA 11.0 College Station, TX and R software Bioconductor package. The clustering was built using heatmap from made4 package. Survival functions were estimated using the Kaplan-Meier method. Difference was tested by log-rank test.

## Supporting Information

Figure S1 **Basal morphology of lung adenocarcinoma cell lines.** H1650, H1975 and HCC827 are *EGFR* mutated, H358 and H1299 are *EGFR* wild type. Representative photomicrograph of cells was obtained by phase-contrast microscopy.
(TIF)

Figure S2 **Impact of EGF removal on EMT markers in H1650.** Image shows the modification of cell morphology up on EGF removal. Graphs show the evolution of EMT marker expression up on EGF removal in H1650 cells by quantitative RT-PCR. mRNA levels are expressed relative to the untreated control condition.
(TIF)

Figure S3 **Impact of TGFβ treatment on EMT markers in HCC827.** HCC827 cells were treated with TGFβ or EGF during 24, 48 h and 10 days at 25 ng/mL. **A.** After treatment, representative photomicrograph of cells was obtained by phase-contrast microscopy. **B.** Expression of *TWIST1*, *CDH2*, *VIM*, *CDH1* and *JUP* was measured by quantitative RT-PCR, after TGFβ or EGF treatments. Each column represents the mean ±SD of 9 wells and three independent experiments.
(TIF)

Figure S4 **ZEB1, SNAI1 and SNAI2 in EGFR mutated cell lines.** Summary of mRNA regulation after EGFR treatment in three *EGFR* mutated cell lines. (−): down-regulation, (+) up-regulation, (/) no difference according to non tumor reference. Detailed information for H1650 (*EGFR* mutated/TWIST1 reactivated) is shown (A) up-regulation of *ZEB1* at RNA level at 4 and 24 h treatment, (B) transitory up-regulation of SNAI1 at 4 h treatment (C) up regulation of SNAI2 at protein level WB and IF experiments.
(TIF)

Table S1 **Summary of tumor samples characteristics.** Description of the tumor samples series in terms of clinico-pathological features, gene mutation (*EGFR*, *TP53*,*KRAS*, *BRAF*, *ERBB2*, *PIK3CA*, *STK11 and AKT1*) as well as *CDKN2A* gene copy number.
(PDF)

Table S2 **Primer sequence for quantitative RT-PCR analyses.**
(PDF)
