# Supplementary figures and images for: TWIST1 a New Determinant of Epithelial to Mesenchymal Transition in EGFR Mutated Lung Adenocarcinoma
Source: PLoS One. 2012 Jan 17;7(1):e29954. doi: 10.1371/journal.pone.0029954 (PMC3260187; doi:10.1371/journal.pone.0029954)

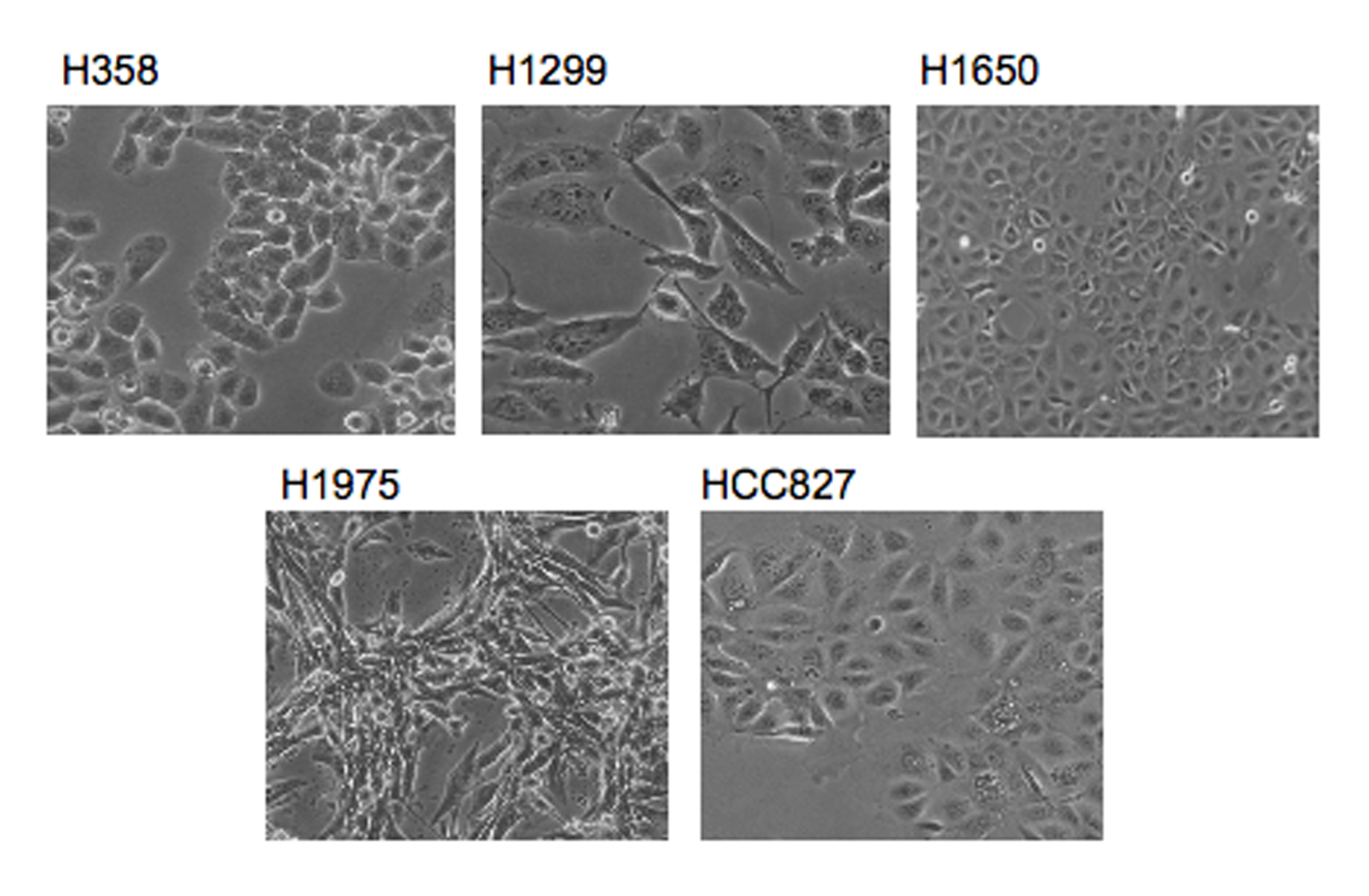

Supplement: Figure S1 — Basal morphology of lung adenocarcinoma cell lines. H1650, H1975 and HCC827 are EGFR mutated, H358 and H1299 are EGFR wild type. Representative photomicrograph of cells was obtained by phase-contrast microscopy. (TIF) [file pone.0029954.s001.tif]

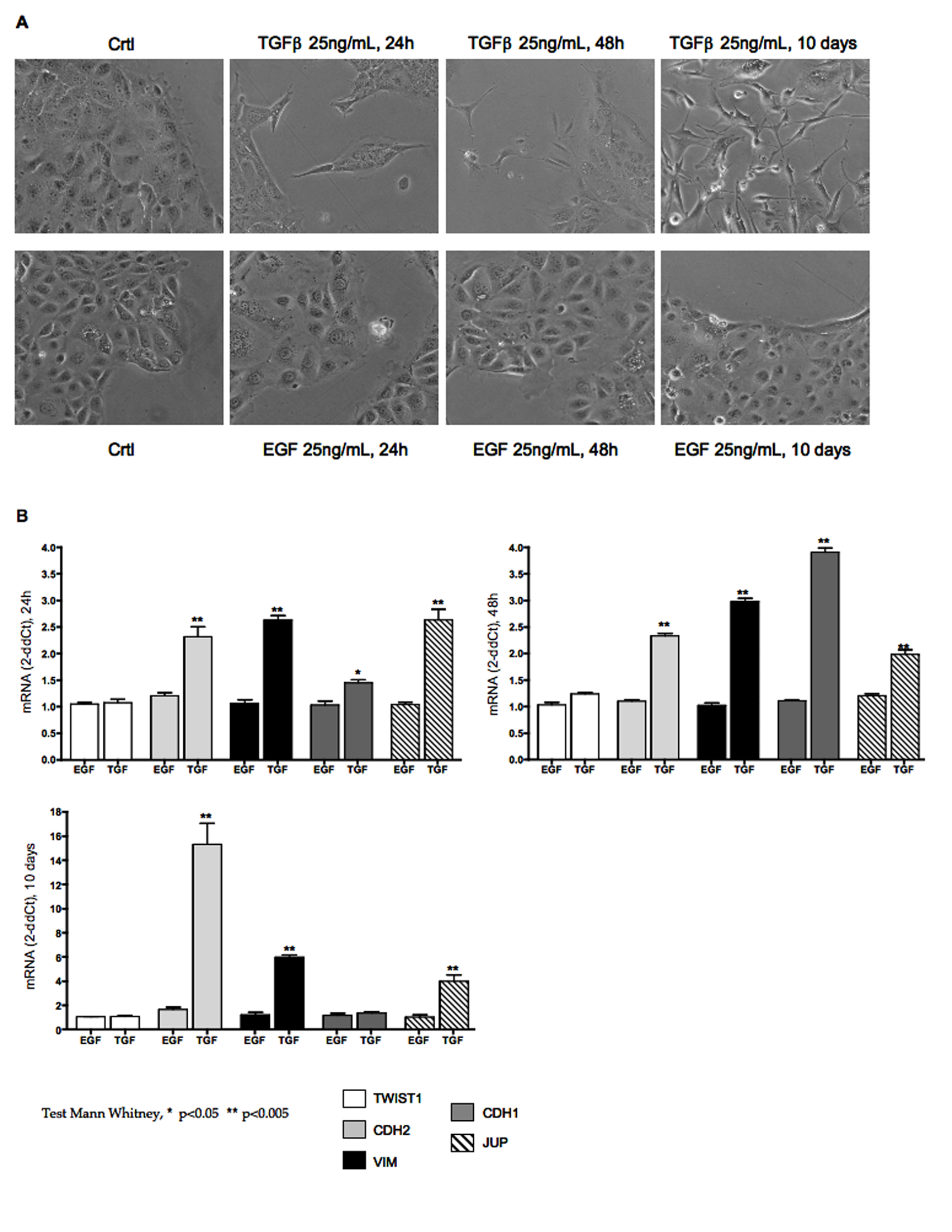

Supplement: Figure S2 — Impact of EGF removal on EMT markers in H1650. Image shows the modification of cell morphology up on EGF removal. Graphs show the evolution of EMT marker expression up on EGF removal in H1650 cells by quantitative RT-PCR. mRNA levels are expressed relative to the untreated control condition. (TIF) [file pone.0029954.s002.tif]

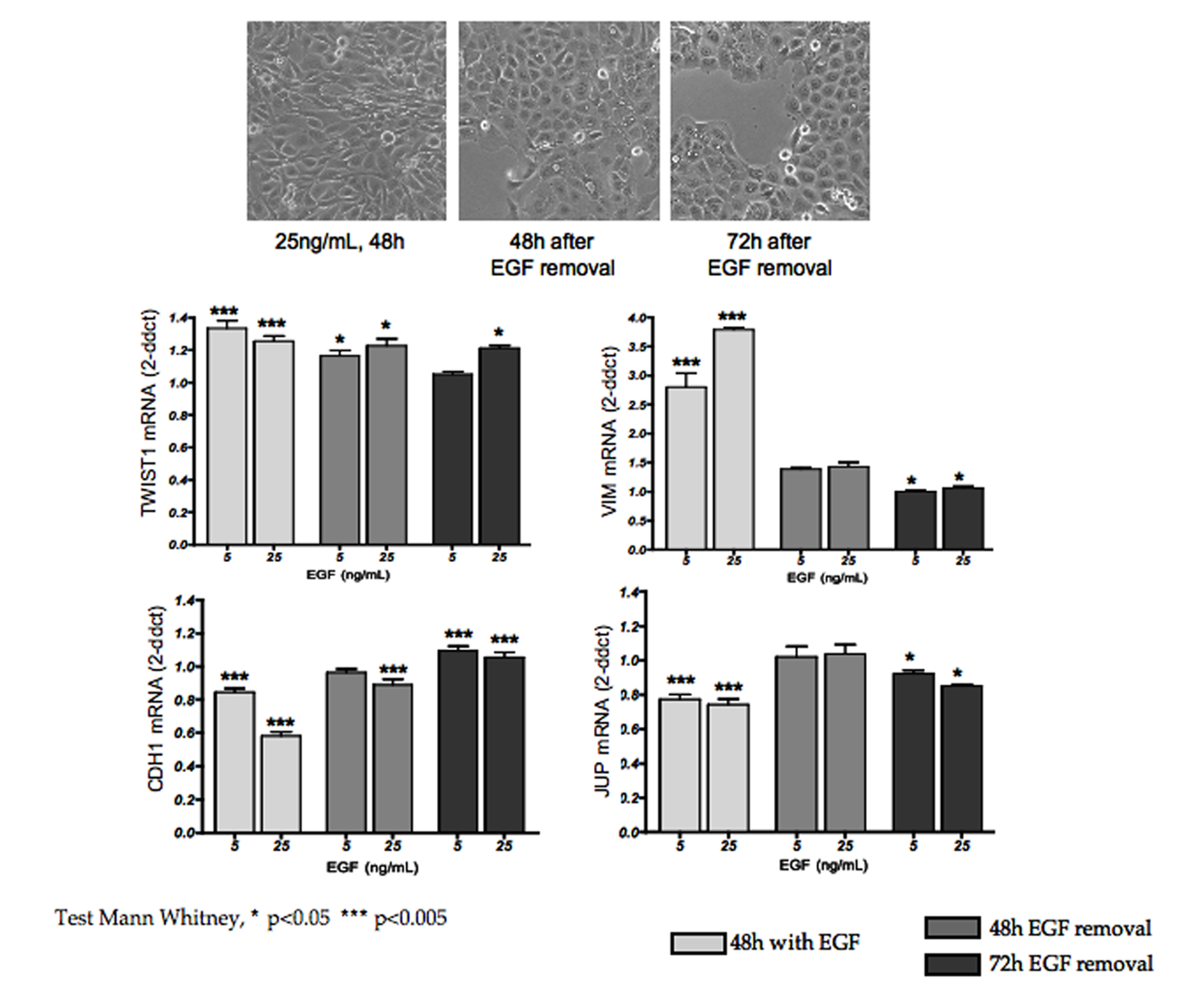

Supplement: Figure S3 — Impact of TGFβ treatment on EMT markers in HCC827. HCC827 cells were treated with TGFβ or EGF during 24, 48 h and 10 days at 25 ng/mL. A. After treatment, representative photomicrograph of cells was obtained by phase-contrast microscopy. B. Expression of TWIST1, CDH2, VIM, CDH1 and JUP was measured by quantitative RT-PCR, after TGFβ or EGF treatments. Each column represents the mean ±SD of 9 wells and three independent experiments. (TIF) [file pone.0029954.s003.tif]

|             | TWIST1 | SNAI1 | ZEB1 | CDH2 | VIM | CDH1 | JUP |
|-------------|--------|-------|------|------|-----|------|-----|
| H1650       | +      | /     | -    | /    | -   | +    | +   |
| H1650 + EGF | ++     | +     | +    | /    | +++ | -    | -   |

  

|             |   |   |   |   |   |   |   |
|-------------|---|---|---|---|---|---|---|
| H1975       | - | + | - | - | / | + | / |
| H1975 + EGF | - | + | - | - | / | + | + |

  

|              |   |   |   |   |   |   |   |
|--------------|---|---|---|---|---|---|---|
| HCC827       | - | - | - | / | - | + | / |
| HCC827 + EGF | - | - | - | / | - | + | / |

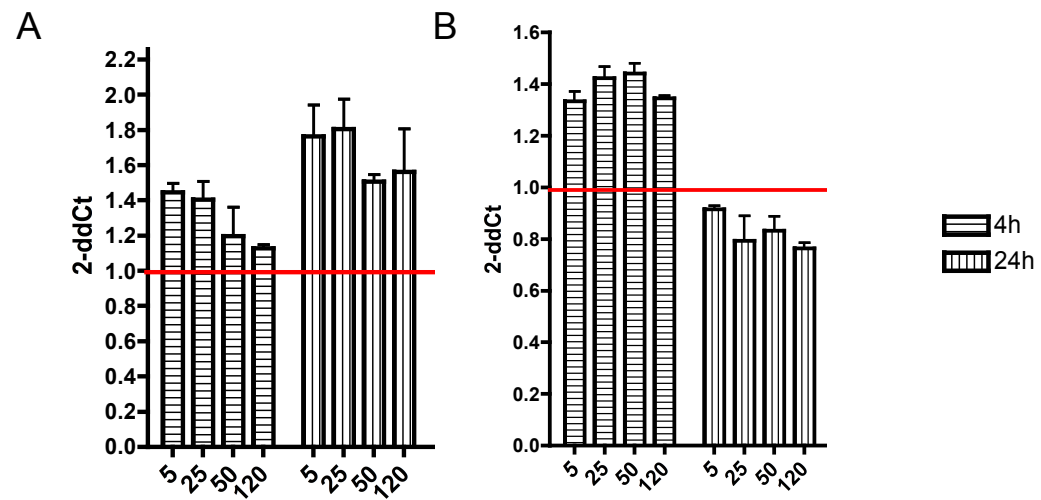

**C**

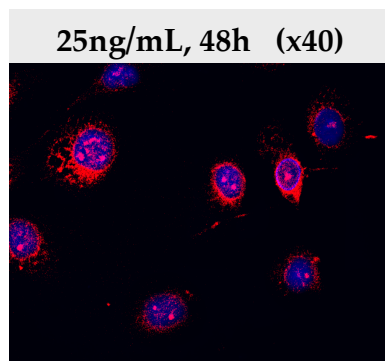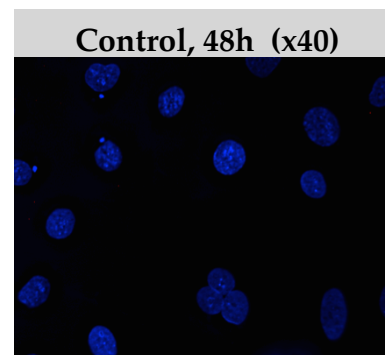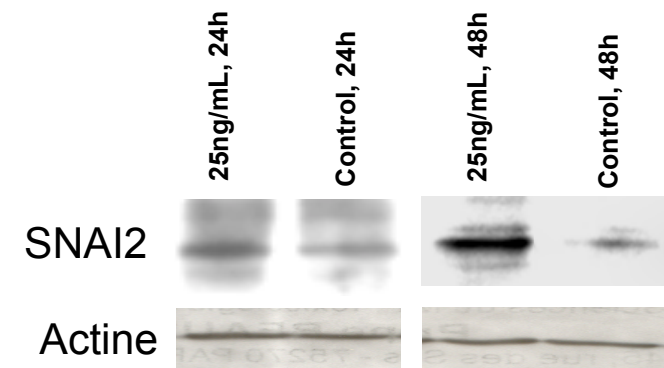

Supplement: Figure S4 — ZEB1, SNAI1 and SNAI2 in EGFR mutated cell lines. Summary of mRNA regulation after EGFR treatment in three EGFR mutated cell lines. (−): down-regulation, (+) up-regulation, (/) no difference according to non tumor reference. Detailed information for H1650 (EGFR mutated/TWIST1 reactivated) is shown (A) up-regulation of ZEB1 at RNA level at 4 and 24 h treatment, (B) transitory up-regulation of SNAI1 at 4 h treatment (C) up regulation of SNAI2 at protein level WB and IF experiments. (TIF) [file pone.0029954.s004.tif]
