# Supplementary material for: TWIST1 a New Determinant of Epithelial to Mesenchymal Transition in EGFR Mutated Lung Adenocarcinoma
Source: PLoS One. 2012 Jan 17;7(1):e29954. doi: 10.1371/journal.pone.0029954 (PMC3260187; doi:10.1371/journal.pone.0029954)
Supplement: Table S1 — Summary of tumor samples characteristics. Description of the tumor samples series in terms of clinico-pathological features, gene mutation (EGFR, TP53,KRAS, BRAF, ERBB2, PIK3CA, STK11 and AKT1) as well as CDKN2A gene copy number. (PDF) [file pone.0029954.s005.pdf]

Table S1. Summary of tumor samples characteristics

Description of the tumor samples series in terms of clinico-pathological features, gene mutation (*EGFR*, *TP53*, *KRAS*, *BRAF*, *ERBB2*, *PIK3CA*, *STK11* and *AKT1*) as well as *CDKN2A* gene copy number.

Clinical and pathological parameters						Protein expression	mRNA expression								Gene mutation								Gene copy number
**Tumour**	**Gender**	**Age**	**Smoker**	**Histology**	**TNM**	**TWIST1 (IHC)**	**RQI**	**TWIST1**	**CDH2**	**CDH1**	**JUP**	**SNAI1**	**ZEB1**	**VIM**	**EGFR**	**TP53**	**KRAS**	**BRAF**	**ERBB2**	**PIK3CA**	**STK11**	**AKT1**	**CDKN2A (2*2-ddCt)**
10	F	79	Non smoker	ADC-BAC	T4N0	Negative	mRNA not available								**Yes**	No	No	No	No	No	No	No	0,17
17	F	84	Non smoker	ADC-BAC	T2N0	**Positive**	7,1	**24,01**	0,95	0,33	0,19	0,45	ND	0,19	**Yes**	No	No	No	No	No	No	No	0,45
18	F	65	Non smoker	ADC-BAC	T2N0	Negative	mRNA not available								**Yes**	Yes	No	No	No	No	No	No	1,09
29	F	61	Non smoker	ADC	T2N1	Negative	mRNA not available								**Yes**	No	No	No	No	No	No	No	0,15
30	F	59	Non smoker	ADC-BAC	T4N2	Negative	mRNA not available								**Yes**	Yes	No	No	No	No	No	No	0,30
31	F	75	Non smoker	ADC-BAC	T2N2	Negative	mRNA not available								**Yes**	No	No	No	No	No	No	No	0,51
39	F	75	Non smoker	ADC-BAC	T2N0	Negative	mRNA not available								**Yes**	Yes	No	No	No	No	No	No	0,46
41	F	56	Non smoker	ADC-BAC	T1N0	Negative	mRNA not available								**Yes**	No	No	No	No	No	No	No	0,65
43	F	72	Non smoker	ADC-BAC	T2N0	Negative	mRNA not available								**Yes**	No	No	No	No	No	No	No	0,29
49	F	75	Non smoker	BAC	T2N0	Negative	mRNA not available								**Yes**	No	No	No	No	No	No	No	0,79
52	F	78	Non smoker	ADC-BAC	T2N2	Negative	8	0,23	2,05	0,16	0,22	0,29	0,31	30,33	No	No	No	No	No	No	No	No	0,90
54	F	78	Non smoker	ADC-BAC	T2N0	**Positive**	9,4	**14,88**	2,27	6,32	3,25	0,47	0,14	0,58	**Yes**	No	No	No	No	No	No	No	0,95
55	F	72	Non smoker	ADC-BAC	T2N2	**Positive**	8,9	**5,91**	0,56	6,35	2,00	0,38	0,71	0,88	**Yes**	Yes	No	No	No	No	No	No	0,86
113	F	46	Non smoker	BAC	T4N0	formalin fixed paraffin not available	6,2	0,03	0,00	0,23	0,38	0,10	0,80	8,83	No	No	No	No	No	No	No	No	1,08
129	F	46	Non smoker	BAC	T4N0	formalin fixed paraffin not available	7,6	0,02	0,00	0,15	0,27	0,10	0,54	24,04	No	No	No	No	No	No	No	No	0,82
130	F	46	Non smoker	BAC	T4N0	formalin fixed paraffin not available	8,4	0,15	0,96	0,28	0,88	0,37	0,35	12,69	No	No	Yes	No	No	No	No	No	0,61
133	F	72	Non smoker	Adsq	T2N0	**Positive**	9,2	**3,14**	6,43	0,04	0,38	0,16	0,59	28,52	No	No	No	No	No	No	No	No	0,98
134	F	62	Non smoker	ADC-BAC	T2N0	**Positive**	9,9	**3,35**	0,57	7,60	1,29	0,25	0,27	0,74	**Yes**	Yes	No	No	No	No	No	No	1,07
135	F	72	Non smoker	BAC	T1N0	Negative	9,2	1,02	0,41	2,46	0,70	0,13	0,36	0,36	**Yes**	No	No	No	No	No	No	No	0,23
140	M	65	Smoker	ADC	T2N0	Negative	7,8	1,59	0,41	2,40	1,01	0,92	0,59	0,51	**Yes**	No	No	No	No	No	No	No	0,86

142	F	64	Non smoker	ADC-BAC	T1N0	Negative	8,1	0,09	5,12	0,14	0,19	0,20	0,53	28,69	No	No	No	No	No	No	No	No	0,89
147	M	83	Non smoker	ADC	T2N1	Negative	8,7	0,08	0,72	0,14	0,68	0,30	0,81	10,66	No	No	No	No	No	No	No	No	1,09
149	M	85	Non smoker	ADC	T1N0	Negative	7,8	0,01	2,14	0,11	0,49	0,06	0,87	7,93	No	No	No	No	No	No	No	No	0,87
170	M	66	Non smoker	ADC-BAC	T2N0	**Positive**	9,6	**2,03**	0,49	3,87	0,63	0,26	0,30	0,57	**Yes**	No	No	No	No	No	No	No	0,56
201	F	56	Non smoker	ADC	T2N?	Negative	9	0,01	0,27	0,13	0,11	0,04	0,48	8,59	No	No	No	No	No	No	No	No	0,69
230	M	59	Smoker	ADC-BAC	T4N0	Negative	8,3	1,33	1,02	2,60	1,14	0,43	0,48	0,44	**Yes**	Yes	No	No	No	No	No	No	0,63
238	M	62	Non smoker	ADC	T4NOM+	Negative	8,5	0,51	9,99	0,10	0,22	0,39	1,13	48,79	No	ND	No	ND	No	ND	ND	ND	ND
244	F	59	Non smoker	ADC-BAC	T1N0	Negative	mRNA not available								**Yes**	No	No	No	No	No	No	No	0,97
245	M	46	Non smoker	ADC	T4N2	Negative	7,3	0,35	3,13	0,07	0,20	1,10	0,94	36,68	No	No	No	No	No	No	No	No	0,61
247	F	75	Smoker	ADC-BAC	T2N0	Negative	7,3	**8,57**	2,68	3,52	0,79	0,64	0,43	0,97	**Yes**	No	No	No	No	No	No	No	0,73
256	M	61	Non smoker	ADC	T2N1	Negative	7,6	0,28	1,33	0,11	0,18	0,13	1,28	25,99	No	No	No	No	No	No	No	No	1,98
288	F	51	Non smoker	ADC	T1N0M+	**Positive**	8,1	**5,44**	4,08	1,75	1,36	3,61	0,94	2,17	**Yes**	No	No	No	No	No	No	No	0,96
341	F	76	Non smoker	ADC-BAC	T2N0	Negative	mRNA not available								**Yes**	No	No	No	No	No	No	No	0,92

TNM: tumor stage defined by Tumor, Node involvement and Metastasis; ADC: adenocarcinoma; BAC: bronchioloalveolar carcinoma; Adsq: adenosquamous carcinoma; IHC: Immuno-Histo-Chemistry; homozygous CDKN2A (P16) deletion is defined by 2*2-ddCt <0.35
