# Supplementary material for: TWIST1 a New Determinant of Epithelial to Mesenchymal Transition in EGFR Mutated Lung Adenocarcinoma
Source: PLoS One. 2012 Jan 17;7(1):e29954. doi: 10.1371/journal.pone.0029954 (PMC3260187; doi:10.1371/journal.pone.0029954)
Supplement: Table S2 — Primer sequence for quantitative RT-PCR analyses. (PDF) [file pone.0029954.s006.pdf]

Table S2: Primer sequence for quantitative RT-PCR analyses

Gene	Primers	
TWIST1	Forward	GGAGTCCGCAGTCTTACGAG
	Reverse	CCAGCTTGAGGGTCTGAATC
POLR2A	Forward	TCTTCTGCTCCAAACTGCTTGTGG
	Reverse	GTCGTAGACATGTGTGAGC
RPL13A	Forward	CATGAGGCTACGGAAACAGGC
	Reverse	AAGGGCAGGCAACGCATGA
YAP1	Forward	CACAGCATGTTCGAGC
	Reverse	GATGCTGAGCTGTGGGTGTA
JUP	Forward	CAGCAGCCCTACACGGATGGTGTGA
	Reverse	CGGTTCATGGGGTCCCGGGC
VIM	Forward	ATCCAAGTTTGCTGACCTCTCTGAG
	Reverse	AGGGACTGCACCTGTCTCCGGT
CDH1	Forward	GGAACTATGAAAAGTGGGCTTG
	Reverse	AAATTGCCAGGCTCAATGAC
CDH2	Forward	CTTGTCAGGATCAGGTCT
	Reverse	GAAGATACCAGTTGGAGGCT
SNAIL	Forward	CTTCCAGCAGCCCTACGAC
	Reverse	CGGTGGGGTTGAGGATCT
ZEB1	Forward	ACTGCTGGGAGGATGACAGA
	Reverse	ATCCTGCTTCATCTGCCTGA
18S	Forward	TCCCCCAACTTCTTAGAGG
	Reverse	CTTATGACCCGCACTTACTG
